# Targeting p110gamma in gastrointestinal cancers: attack on multiple fronts

**DOI:** 10.3389/fphys.2014.00391

**Published:** 2014-10-15

**Authors:** Marco Falasca, Tania Maffucci

**Affiliations:** Inositide Signalling Group, Blizard Institute, Barts and The London School of Medicine and Dentistry, Queen Mary University of LondonLondon, UK

**Keywords:** angiogenesis, cancer, HCC, inflammation, metastasis, p110γ, PDAC, phosphoinositide 3-kinase

## Abstract

Phosphoinositide 3-kinases (PI3Ks) regulate several cellular functions that are critical for cancer progression and development, including cell survival, proliferation and migration. Three classes of PI3Ks exist with the class I PI3K encompassing four isoforms of the catalytic subunit known as p110α, p110β, p110γ, and p110δ. Although for many years attention has been mainly focused on p110α recent evidence supports the conclusion that p110β, p110γ, and p110δ can also have a role in cancer. Amongst these, accumulating evidence now indicates that p110γ is involved in several cellular processes associated with cancer and indeed this specific isoform has emerged as a novel important player in cancer progression. Studies from our laboratory have identified a specific overexpression of p110γ in human pancreatic ductal adenocarcinoma (PDAC) and in hepatocellular carcinoma (HCC) tissues compared to their normal counterparts. Our data have further established that selective inhibition of p110γ is able to block PDAC and HCC cell proliferation, strongly suggesting that pharmacological inhibition of this enzyme can directly affect growth of these tumors. Furthermore, increasing evidence suggests that p110γ plays also a key role in the interactions between cancer cells and tumor microenvironment and in particular in tumor-associated immune response. It has also been reported that p110γ can regulate invasion of myeloid cells into tumors and tumor angiogenesis. Finally p110γ has also been directly involved in regulation of cancer cell migration. Taken together these data indicate that p110γ plays multiple roles in regulation of several processes that are critical for tumor progression and metastasis. This review will discuss the role of p110γ in gastrointestinal tumor development and progression and how targeting this enzyme might represent a way to target very aggressive tumors such as pancreatic and liver cancer on multiple fronts.

## Introduction

Gastrointestinal cancers comprise a group of cancers that affect the gastrointestinal tract and include esophageal, stomach, liver, pancreatic and colorectal cancer. Gastrointestinal cancers have one of the poorest prognoses among all cancers partly because of their silent nature and tendency for late discovery but also because of their peculiar resistance to chemotherapy and radiation therapy (Lockhart et al., 2005; Schneider et al., 2005; Thrumurthy et al., 2013; Zhang, 2013; Brenner et al., 2014; Singh et al., 2014). The prospect for patients with liver and pancreatic cancer is particularly dismal. For instance, the 5 year survival rate for pancreatic ductal adenocarcinomas (PDAC) is the lowest among all cancers (Li et al., 2004). Similarly in 2011 hepatocellular carcinoma (HCC) was estimated as the second and sixth leading cause of cancer-related death in men and women respectively (Jemal et al., 2011). There have been great advances in survival rates for many types of cancers over the past few decades but hardly any change for PDAC and HCC. There are very few treatments for PDAC, most of them just palliative. Similarly only a small percentage of patients with HCC are eligible for surgery since the majority of patients present with advanced or unresectable disease (Kuper et al., 2000; Thomas et al., 2010; Singh et al., 2014). For many years systemic chemotherapy also proved to be only minimally effective (Ryder, 2003; Thomas et al., 2008) and currently only the multikinase inhibitor sorafenib is approved for advanced HCC patients (Thomas et al., 2010). There is therefore an urgent need to better understand the mechanisms underlying progression of these cancer types in order to develop novel potential chemotherapeutic agents.

It is well known that mutations in *K-Ras* oncogene occur in 75–90% of PDAC and accumulate early in the disease progression (Moskaluk et al., 1997; Hruban et al., 2000). On the other hand, metastatic colorectal cancer represents one of the largest hurdles in cancer treatment and metastatic colorectal tumors with a mutation in *K-Ras* do not respond to available treatments such as anti-epidermal growth factor receptor monoclonal antibodies (Brand and Wheeler, 2012). K-Ras signaling promotes the neoplastic phenotype via activation of downstream targets that control membrane trafficking, cellular proliferation, differentiation and cytoskeleton organization (Lockhart et al., 2005). A key downstream target of the Ras family is phosphoinositide 3-kinase (PI3K), the enzyme responsible for generation of 3-phosphorylated phosphoinositides and activation of the protein kinase B/Akt (Kodaki et al., 1994; Chang et al., 1997; Khwaja et al., 1997; Luo et al., 2003). Indeed activation of Akt has been observed in PDAC and represents a biological indicator of the aggressiveness of the disease (Yamamoto et al., 2004). Akt and in particular its downstream effector mechanistic target of rapamycin (mTOR) have also been established as key molecular targets in HCC and inhibitors of these molecules have been tested in clinical trials (Shen et al., 2013). Although eight distinct PI3K isoforms exist most of the studies on PI3K and cancer have been focused so far on one specific isoform, p110α that has been found to be mutated in several cancer types. Only recently increasing evidence has suggested that other PI3K isoforms may also play a non-redundant role in different tumor settings. The aim of this review is to summarize the evidence indicating that the PI3K isoform p110γ plays a key role in gastrointestinal cancers.

## Phosphoinositide 3-kinases

PI3Ks catalyze the phosphorylation of lipids known as phosphoinositides in position 3 of their inositol rings (Falasca and Maffucci, 2012). Phosphatidylinositol 3,4,5-trisphosphate [PtdIns(3,4,5)*P*_*3*_], originally identified in activated neutrophils from human donors (Traynor-Kaplan et al., 1988), is the best characterized of the PI3K lipid products and it plays a key role in activation of several signaling molecules. These include Akt, 3 phosphoinositide dependent protein kinase 1 and their effector proteins such as mTOR that in turn regulate signaling cascades involved in cell growth, survival, proliferation, motility and morphology. It is now well-established that the PI3K/Akt pathway plays a pivotal role in several processes that are critical for cancer development and progression including inhibition of apoptosis, stimulation of cell proliferation and drug resistance (Luo et al., 2003; Takeda et al., 2004). Indeed it has been estimated that at least 50% of all cancer types present some deregulation of this signaling pathway (Yuan and Cantley, 2008). In particular, the PI3K/Akt pathway is activated in almost 60% of PDAC (Bondar et al., 2002; Schlieman et al., 2003) and it plays a critical role in HCC (Zhou et al., 2011).

Eight PI3K isoforms exist in mammalian cells and they have been grouped into three classes according to their structure and substrate specificity (Vanhaesebroeck et al., 2001; Falasca and Maffucci, 2007, 2012) with the PI3K class I encompassing four isoforms of the catalytic subunit known as p110α, p110β, p110γ, and p110δ. Amongst these, p110α has a well-established role in cancer and gain of function of this isoform due to mutation of its gene *PIK3CA* is common in several human cancers (Samuels et al., 2004; Zhao and Vogt, 2008). Whilst mutations to PI3K are commonly associated with *PIK3CA* it is important to notice that mutations have actually been found in all PI3K isoforms, although their prevalence and functional relevance in disease is considered limited. An overview of these mutations can be found on the COSMIC website (http://www.sanger.ac.uk/genetics/CGP/cosmic/). Although somatic mutations of the genes encoding the other isoforms are less frequent, accumulating data now suggest that p110β, p110γ, and p110δ can also have a role in cancer.

It was previously reported that while overexpression of wild-type p110α does not have transforming potential, overexpression of the wild-type catalytic subunits p110β, p110γ, and p110δ is sufficient to induce an oncogenic phenotype in cultured cells (Kang et al., 2006). These data suggested that increased expression levels of the “non α” catalytic subunits rather than gain of function mutations can be relevant in cancer development and progression. Indeed increased levels of both p110β and p110δ have been observed in glioblastoma and in some colon and bladder tumors (Bénistant et al., 2000; Knobbe and Reifenberger, 2003). p110β has been shown to stimulate cell proliferation and invasive cell growth (Czauderna et al., 2003) whereas p110δ controls proliferation in acute myeloid leukemia (Sujobert et al., 2005) and migration of breast cancer cells (Sawyer et al., 2003). More recently it has been reported that inactivation of p110δ in mice models inhibits different cancer types and induces tumor regression (Ali et al., 2014). Interestingly, amongst the distinct cancer models investigated in this study, the authors also reported that treatment with PI-3065, a small molecule inhibitor with selectivity for p110δ, prolonged survival and reduced the incidence of macroscopic metastases in the LSL *KRas*^G12D/+^; *p53*^R172H/+^; *Pdx*^Cretg/+^ model of PDAC (Ali et al., 2014).

Accumulating evidence from several groups indicates that the class IB isoform p110γ has a role in several cellular processes involved in tumor development and progression, including proliferation of pancreatic and liver cancer cells (Edling et al., 2010; Dituri et al., 2012), tumor angiogenesis (Hamada et al., 2005), drug resistance in chronic myeloid leukemia cells (Hickey and Cotter, 2006) and many more processes. This review will discuss the current evidence supporting the conclusion that inhibition of p110γ can represent an important strategy to target cancers on multiple fronts.

## p110γ

The PI3K catalytic subunit p110γ is encoded by the *PIK3CG* gene, located on chromosome 7q22.3 and its activity is modulated via interaction with the p101 (*PIK3R5*) and p87 (*PIK3R6*) regulatory subunits (Fyffe et al., 2013). The original classification of this isoform into the class IB subgroup of PI3Ks mainly derived from the observation that p110γ can be activated downstream of G-protein coupled receptors (GPCRs). Evidence however suggests that tyrosine kinase receptors (RTKs) can also activate p110γ (Ptasznik et al., 1996; Vanhaesebroeck et al., 2010). Importantly, Ras has also a key role in p110γ activation.

p110γ is mainly expressed in hematopoietic cells and is involved in immune, inflammatory and allergic responses (Vanhaesebroeck et al., 2010). Several studies have indicated a key role for this isoform in migration of hematopoietic cell types. For instance it has been reported that p110γ modulates leukocyte chemotaxis to inflammatory sites and in response to chemoattractant agents and it is also involved in motility of dendritic cells (Del Prete et al., 2004). Both p110γ and p110δ are involved in natural killer cell development and migration toward the sites of inflammation and in T-lymphocyte migration and development (So and Fruman, 2012). In addition to a specific role in migration, p110γ can also regulate T-lymphocyte proliferation and cytokine production (So and Fruman, 2012). It has also been demonstrated that combined inactivation of p110γ and p110δ impairs B cell development and reduces B cell numbers to a greater extent than p110δ inactivation alone (Beer-Hammer et al., 2010). The lipid kinase activity of the enzyme seems to be important for regulation of these processes and indeed p110γ could play a role in leukocyte polarization and migration by regulating the spatial accumulation of PtdIns(3,4,5)*P*_*3*_, the organization of F-actin formation and integrin-based adhesion at the leading edge (Hirsch et al., 2000). Interestingly it has been demonstrated that p110γ also possesses a serine/threonine protein kinase activity that is independent from its lipid kinase activity (Hirsch et al., 2001). Specifically it has been reported that p110γ plays a key role in platelet aggregation and thrombosis by regulating αIIb/β3 integrin adhesive function in platelets downstream of P2Y12 through a mechanism that is independent from its lipid kinase activity (Hirsch et al., 2001). In addition it has been reported that p110γ can control cardiac contractility through formation of a multiprotein complex with PDE3B and independently from its kinase activity (Patrucco et al., 2004). Taken together these data indicate that p110γ can be involved in several cellular functions through distinct molecular mechanisms.

## Role of p110γ in cancer cell proliferation

Although p110γ is primarily expressed in leukocytes and has a well characterized role in immunity (Hirsch et al., 2014) evidence also indicates a role for this PI3K isoform in some cancer types. It must be noted that an original investigation reported that p110γ^−/−^ mice on 129J background developed macroscopically visible tumors mainly at the proximal and distal parts of the large intestine (Sasaki et al., 2000). However, the authors did not observe the same tumor phenotype after backcrossing these mice onto a C57BL/6 background, neither they observed formation of tumors when they retargeted the allele in different ES cells using the same targeting construct. Therefore, the authors changed their original conclusion that inactivation of p110γ leads to development of invasive colorectal adenocarcinomas in mice (Sasaki et al., 2000) by stating that this inactivation does not in itself cause colon cancer (corrigenda Sasaki et al., 2003).

Although the prevalence and functional relevance of mutations in PI3Ks other than p110α are considered limited it is worth mentioning that significant recurrent mutations have been seen in both *PIK3CG* (9.7%) and *PIK3C2B* (12.9%), the gene encoding for the class II isoform PI3K-C2β, in lung cancer (Liu et al., 2012). Interestingly *PIK3CG* is located in a region of chromosome band 7q22 that is frequently deleted in myeloid malignancies.

Recently, new driver mutations in pancreatic cancer have been identified using cancer-specific high-throughput annotation of somatic mutations (Carter et al., 2010). Importantly, *PIK3CG* contains the second highest scoring predicted driver mutation among the set of genes not previously identified as a driver in pancreatic cancer. The specific *PIK3CG* mutation identified is R839C and the residue Arg 839 is located within the C-terminal catalytic domain. Although the authors suggest that this mutation may lead to a loss of function of the enzyme based on X-ray crystal structure (Carter et al., 2010) it actually remains to be established whether it indeed results in modulation of p110γ catalytic activity and the potential functional consequences on pancreatic cancer cells.

Recent studies in our laboratory have identified a selective accumulation of p110γ in specific cancer types. In particular, in an extensive investigation of the expression of all PI3Ks we detected a specific overexpression of p110γ in PDAC tissues compared to normal counterparts (Edling et al., 2010). Similarly immunohistochemistry analysis of PI3Ks in HCC and paired peritumoral human tissues showed p110γ expression in HCC tissues, in particular in epithelial cancer cells (Dituri et al., 2012). Importantly p110γ correlated with the proliferative marker Ki-67 in these tissues, indicating accumulation of this isoform in cancer cells with high proliferative index (Dituri et al., 2012). Consistent with this, our data demonstrated that p110γ is required for PDAC and HCC cell proliferation. Specifically we reported that chemical inhibition as well as selective siRNA-mediated downregulation of p110γ reduced pancreatic cancer cell growth without increasing cell apoptosis (Edling et al., 2010). Similarly we showed that downregulation of p110γ in HCC cell lines specifically induced arrest of cell cycle in the G2/M phase through modulation of p21 levels (Dituri et al., 2012). These data indicate that p110γ has a specific role in regulation of cancer cell proliferation and strongly suggest that overexpression of p110γ has a functional role in progression of PDAC and HCC. The molecular mechanisms regulating p110γ overexpression in specific cancer types are yet to be established.

Taken together these data suggest that inhibition of p110γ can inhibit proliferation of specific cancer types. In this respect we recently demonstrated that treatment with the caffeine analog CGS 15943 inhibited proliferation of HCC and PDAC cell lines (Edling et al., 2014). Interestingly, a kinase profiler analysis revealed that CGS 15943 is able to inhibit p110γ (Edling et al., 2014), further supporting the conclusion that targeting this PI3K isoform may prove beneficial to directly reduce PDAC and HCC growth (Falasca et al., 2011).

## Role of p110γ in cancer metastasis

Metastasis, the ability of cancer cells to spread from a primary site and form tumors at distant sites, is the main cause of death of most cancer patients. Several steps regulate the development of metastasis including migration of cancer cells out of the primary tumor, local invasion, intravasation into the circulatory system, survival, extravasation, initiation and maintenance of micro-metastases at distant sites and vascularization of the resulting tumors (Nguyen and Massague, 2007). A peculiar feature of this process is the variability in metastatic tissue tropism shown by different types of cancer. Indeed, even though cancer cells can spread to almost every area of the body, each cancer type shows specific preference for common regions where cancer may spread to (Nguyen et al., 2009). Cancer cells with high metastatic potential are characterized by high proliferation and migration. The endogenous signaling pathways associated with high metastatic potential remain unclear.

Although it was well established that p110γ is involved in the regulation of migration of different cell types, including leukocytes and endothelial cells (ECs) (as discussed below) only recently a specific role for this isoform in regulation of cancer cell dissemination and metastasis has been suggested (Attoub et al., 2008; Brazzatti et al., 2012; Xie et al., 2013). Specifically it has been reported that shRNA-mediated downregulation of p110γ reduces the ability of breast cancer cells MDA-MB-231 to metastasize *in vivo* (Brazzatti et al., 2012). Similar data were obtained in the 4T1.2 mouse model of breast cancer (Brazzatti et al., 2012). Several mechanisms of p110γ-dependent regulation of metastasis formation have been proposed. For instance recent data indicate that p110γ exerts transforming functions via several mechanisms in human colon epithelial cancer cells, including alteration of homotypic cell–cell adhesion and induction of collagen type I invasion through canonical pro-invasive pathways (Attoub et al., 2008). The pro-metastatic activity of p110γ in metastatic epithelial carcinoma cells seems to involve its ability to prevent anoikis, an apoptotic process resulting from disruption of cell-matrix interaction-dependent survival (Brazzatti et al., 2012). Evidence also suggests that p110γ can be directly involved in cancer cell migration and invasion, as it has been shown in MDA-MB-231 (Brazzatti et al., 2012; Xie et al., 2013) and in melanoma cells (Monterrubio et al., 2009) in response to the chemokine CXCL12. In addition, p110γ is involved in lysophosphatidic acid (LPA) signaling (Edling et al., 2010). LPA signaling has been shown to play a role in cancer and in particular in cancer cell migration (Van Meeteren and Moolenaar, 2007). Both CXCL12 and LPA are key regulators of metastatic processes in several cancers including gastrointestinal cancers. Interestingly CXC chemokine receptor 4 plays an important role in pancreatic cancer metastasis, and initiates G-protein signaling when activated by its ligand CXCL12 (Marchesi et al., 2004).

Taken together these data support the conclusion that inhibition of p110γ can affect cancer cell migration and metastasis formation.

## Roles of p110γ in angiogenesis

A prerequisite for tumor growth is the supply of nutrients and oxygen that are provided by the formation of new blood vessels in a process named tumor angiogenesis (Welti et al., 2013). Several cancer types, including HCC, strongly rely on tumor angiogenesis and anti-angiogenic strategies are either in use or are currently being tested in clinical trials as anti-cancer strategies in several tumor settings.

Tumor angiogenesis involves the coordinated action of different cell types that are normally present or are recruited to the tumor site. In particular tumor angiogenesis is the results of proliferation, migration and remodeling of activated ECs that eventually form novel capillaries (Welti et al., 2013). Several transgenic mouse models have established the pivotal role for class I PI3K isoforms in EC functions. Specifically a central role for p110α has been demonstrated by the observation that knockout mice for this isoform die during embryogenesis (E9.5) due to defects in vasculogenesis and this was confirmed by knock-in strategies (Graupera et al., 2008). Transgenic mouse models further indicated specific roles for p110γ in ECs. Indeed, although knock out and knock in p110γ mice were mainly characterized by immunological and cardiac (in the case of p110γ^−/−^ mice) defects (Morello et al., 2009) it was also observed that muscles from p110γ^−/−^ mice presented reduced capillarization and arteriogenesis following unilateral limb ischemia compared to wild type mice which in turn resulted in delayed blood flow recovery (Madeddu et al., 2008). A role in proliferation and survival of ECs was suggested by the observation that the number of BrdU-positive capillaries was reduced in p110γ^−/−^ ischemic gastrocnemius muscles compared to wild type whereas the percentage of apoptotic capillaries was increased. Interestingly, evidence also suggested that the lipid kinase activity of p110γ was not required in regulation of these cellular processes since post-ischemic neovascularization was not impaired and microvascular apoptosis was not increased in p110γ^KD/KD^ (kinase dead) mice. Furthermore, a defect in proliferation, survival and migration induced by stromal cell-derived factor-1 was detected in endothelial progenitor cells from p110γ^−/−^ but not from p110γ^KD/KD^ mice, suggesting that p110γ may regulate endothelial progenitor cells function and muscular angiogenesis through a mechanism independent of its kinase activity. On the other hand both p110γ^−/−^ and p110γ^KD/KD^ endothelial progenitor cells showed reduced integration into endothelial networks, indicating that the lipid kinase activity is required for some steps of capillary formation.

Evidence from several groups, including our own, has also identified a role for p110γ in regulation of EC migration. Specifically a previous study demonstrated that both p110β and p110γ are involved in sphingosine 1-phosphate (S1P)-mediated EC migration (Heller et al., 2008). We further reported that chemical inhibition and siRNA-mediated downregulation of p110γ reduced S1P-induced as well as high density lipoprotein 3-induced EC migration (Tibolla et al., 2013). Importantly downregulation of p110γ also inhibited S1P- and high density lipoprotein 3-induced remodeling of ECs on Matrigel indicating a key role for this isoform in regulation of capillary tube formation (Tibolla et al., 2013). Interestingly, analysis of cell motility further indicated a specific role for p110γ in regulation of cell speed and provided direct evidence that different PI3Ks are involved in EC tubule formation.

These data support the conclusion that inhibition of p110γ can reduce EC remodeling and formation of novel capillaries network and can therefore possibly represent a novel strategy to counteract tumor angiogenesis.

## Roles of p110γ in stroma cells

Through generation of lipid second messengers and activation of several signaling molecules PI3Ks regulate several cellular functions, such as cell growth, survival, cytoskeletal remodeling and trafficking of intracellular organelles in many different cell types. The generation of transgenic mice has further revealed a critical role for some isoforms in regulation of the immune system *in vivo* in particular p110δ and p110γ that are preferentially expressed in immune cells.

It is now well-established that the immune system plays an important role during tumorigenesis (Sun and Karin, 2014). Deregulated cell proliferation and survival, genome destabilization and induction of migration and invasion (Elinav et al., 2013) as well as different steps involved in metastasis development (Antonioli et al., 2013) have all been associated with inflammation. For instance invasion and intravasation of cancer cells into blood and lymphatics vessels can be regulated by tumor-associated macrophages and inflammatory factors that can increase vascular permeability, prostaglandin production and matrix metalloproteinases-mediated tissue remodeling (Quail and Joyce, 2013).

Infiltrative macrophages and inflammatory cells have been detected in PDAC stroma which has been intensely investigated in recent years. Indeed PDAC is characterized by desmoplasia deriving from pancreatic stellate cells that proliferate and produce a dense extracellular matrix consisting of collagen, laminin and fibronectin (Apte et al., 2013). Although several lines of evidence support the conclusion that interaction between pancreatic stellate cells and PDAC cells plays a critical role in the disease (Haqq et al., 2014) this information has not been translated into efficient therapeutic strategies yet. In fact while preclinical studies indicated potential benefit in targeting the stroma-associated Hedgehog signaling pathway corresponding clinical trials yielded disappointing results. A renewed interest in understanding the cause of this failure has recently revealed a protective role for the stroma against PDAC (Lee et al., 2014; Özdemir et al., 2014; Rhim et al., 2014) indicating that alternative strategies must be investigated (Gore and Korc, 2014). HCC progression is also frequently associated with continuous hepatocyte death and inflammatory cell infiltration (He and Karin, 2011).

Several lines of evidence suggest that p110γ plays a central role in cancer-associated inflammation. For instance, in a murine model of ulcerative colitis, it has been shown that p110γ regulates the innate immune system by controlling colon inflammation and tumor formation (Gonzalez-Garcia et al., 2010). In this model, p110γ-deficient mice and control mice were treated with dextran sulfate sodium to induce chronic colitis and colitis-associated cancer. The results showed that p110γ-deficient mice had a lower incidence of colitis-associated tumors as well as reduced tumor multiplicity. The reduced tumor development was a consequence of defective infiltration and activation of myeloid cells and defective recruitment of T cells to the colon and less colon inflammation.

It has been recently reported that a range of chemoattractants able to activate GPCRs, RTKs and Toll-like/IL-1 receptors unexpectedly initiate tumor inflammation by activating p110γ in Gr1+CD11b+ myeloid cells (Schmid et al., 2011). While GPCRs activate p110γ in a Ras/p101 dependent manner, RTKs and Toll-like/IL-1 receptors directly activate p110γ in a Ras/p87-dependent manner. Once activated, p110γ promotes inside-out activation of a single integrin, α4β 1, causing myeloid cell invasion in different tumor types including pancreatic cancer (Schmid et al., 2011). Pharmacological or genetic blockade of p110γ suppressed inflammation, growth and metastasis of implanted and spontaneous tumors, indicating that targeting p110γ can further affect tumor progression through its effect on stroma cells and reduction of inflammation.

## Roles of p110γ in cancer metabolism

To meet their increasing demand of building block molecules, cancer cells switch to a heavily glucose-dependent metabolism (Ward and Thompson, 2012). Alteration of metabolic pathways is very common in cancer cells and indeed reprogramming of metabolism has been recently recognized as an emerging hallmark of cancer (Hanahan and Weinberg, 2011). In addition to altered glucose metabolism it is becoming increasingly evident that alteration of lipid metabolism plays also a critical role in cancer development. On the other hand, altered cell metabolism, as observed during obesity and insulin resistant conditions, is becoming increasingly associated with cancer development and progression. Indeed, accumulating evidence now suggests that obesity is associated with increased frequency of several cancer types, including prostate, kidney, esophagus, breast, endometrium cancers, as well as cancers of the stomach, colon, pancreas, gallbladder and liver (Møller et al., 1994; Wolk et al., 2001; Calle et al., 2003; Renehan et al., 2008; Basen-Engquist and Chang, 2011). For instance a recent analysis, with a follow up of 239,597 participants and 897 pancreatic cancer deaths, has indicated that obesity is independently associated with increased pancreatic cancer mortality in African Americans (Bethea et al., 2014). These results are consistent with a recent study of two large prospective cohorts comprising 902 patients with pancreatic cancer that also reported statistically significantly reduced survival in patients with higher body mass index before diagnosis (Yuan et al., 2013). Similarly several studies have indicated a link between obesity and increasing incidence of HCC (Møller et al., 1994; Wolk et al., 2001; Borena et al., 2012; Schlesinger et al., 2013; Turati et al., 2013; Karagozian et al., 2014). For instance a prospective cohort of the Cancer Prevention Study with more than 900,000 North American subjects revealed that men with body mass index ≥35 kg/m^2^ had a higher risk of dying from liver cancer (Calle et al., 2003). A similar study involving 362,552 Swedish men also indicated an increased risk of HCC in individuals with body mass index ≥30 kg/m^2^ (Samanic et al., 2006). The mechanisms responsible for this obesity-associated increase in cancer incidence are not completely known. Obesity is associated with a chronic low-grade inflammation, and specific anti-inflammatory interventions may be beneficial for the treatment of type 2 diabetes and other obesity-related diseases. On the other hand obesity is usually associated with insulin resistance and with increased levels of circulating insulin that can in turn promote cell proliferation and survival. Whether obesity *per se* or the associated insulin resistance is ultimately responsible for this increased cancer risk is still a matter of debate.

Consistent with its central role in leukocyte chemotaxis, mast cell degranulation, and EC activation, p110γ has been demonstrated to be critical for obesity-induced inflammation and insulin resistance. It has been shown that mice lacking functional p110γ were protected from insulin resistance, metabolic inflammation, and fatty liver largely because of their leaner phenotype (Becattini et al., 2011; Kobayashi et al., 2011). This phenotype appeared to be a consequence of decreased fat gain as a result of increased energy expenditure despite normal caloric intake. Indeed p110γ action on diet-induced obesity depends on p110γ activity within a non-hematopoietic compartment, where it promotes energetic efficiency for fat mass gain. Importantly, the metabolic modulation by p110γ depends on its lipid kinase activity but it might also involve kinase-independent signaling.

Apart from its role in control of weight gain, data also indicate that p110γ is directly involved in development of obesity-induced insulin resistance. By using murine models of both diet-induced and genetically induced obesity the role of p110γ in the accumulation of tissue macrophages and the development of obesity-induced insulin resistance was examined (Kobayashi et al., 2011). Mice lacking p110γ exhibited improved systemic insulin sensitivity with enhanced insulin signaling in the tissues of obese animals (Kobayashi et al., 2011). In adipose tissues and livers of obese p110γ^−/−^ mice, the numbers of infiltrated pro-inflammatory macrophages were markedly reduced, leading to suppression of inflammatory reactions in these tissues. Furthermore, bone marrow-specific deletion and pharmacological blockade of p110γ also ameliorated obesity-induced macrophage infiltration and insulin resistance. These data suggest that p110γ plays a crucial role in the development of both obesity-induced inflammation and systemic insulin resistance. These data indicate that targeting p110γ can have additional beneficial effects in the context of obesity- and insulin resistance-associated cancers. Based on this evidence it is also tempting to speculate that p110γ inhibition may have a more generic role in modulation of cancer cell metabolism beyond the context of obesity and insulin resistant conditions.

## p110γ specific inhibitors

Consistent with a key role for PI3Ks in cancer development, several inhibitors have been shown to possess anti-cancer activity *in vitro* and *in vivo*. Although PI3Ks and related signaling pathways have been recognized as important therapeutic targets, development of generic PI3K inhibitors has raised some concerns because of the large number of physiological functions that this family of enzymes controls. Many inhibitors of the PI3K pathway have been generated (Falasca, 2010, 2011) that can target either the enzymes themselves or their downstream effectors and these agents can be divided into four major classes: PI3K inhibitors, dual PI3K-mTOR inhibitors, Akt inhibitors and mTOR inhibitors.

PI3K inhibitors can be divided in pan-class I PI3K inhibitors, which target all class I PI3Ks, such as PI-103 and BEZ235 (Raynaud et al., 2007; Maira et al., 2008) and PI3K isoform-specific inhibitors, which specifically target a single PI3K isoform. Since p110δ and p110γ are highly enriched in leukocytes, they are particularly desirable targets for inhibition in the treatment of hematologic malignancies. Indeed among the first isoform-specific PI3K inhibitors developed, the specific p110δ inhibitor IC87114 was used to demonstrate that p110δ could play a role in the pathophysiology of acute myeloid leukemia (Sadhu et al., 2003). Subsequently, TGX-221, an analog of the generic PI3K inhibitor LY294002, and AS252424 were found to selectively inhibit p110β and p110γ respectively (Jackson et al., 2005; Pomel et al., 2006). In the last decade, a number of pharmaceutical companies have reported a wide variety of p110γ inhibitors (Venable et al., 2010), and several X-ray crystal structures with p110γ have been elucidated (Zvelebil et al., 2008). As a consequence, the efficacy of p110γ inhibitors generated has been demonstrated in different biological systems (Venable et al., 2010). However, one of the issues emerged from these studies is the selectivity of compounds tested. On the other hand, an increased interest has emerged in the development of dual p110δ and p110γ inhibitors (Randis et al., 2008). More recently a potent oral p110δ and p110γ inhibitor (IPI-145) has been characterized (Winkler et al., 2013). It has been demonstrated that IPI-145 exerts profound effects on adaptive and innate immunity by inhibiting B and T cell proliferation, blocking neutrophil migration, and inhibiting basophil activation. The therapeutic value of combined p110δ and p110γ blockade was explored, and IPI-145 showed potent activity in collagen-induced arthritis, ovalbumin-induced asthma, and systemic lupus erythematosus rodent models (Winkler et al., 2013). These findings support the hypothesis that inhibition of immune function can be achieved through p110δ and p110γ inhibition, potentially leading to significant therapeutic effects in multiple inflammatory, autoimmune, and hematologic diseases.

Preclinical studies have shown that PI3K inhibition is able to induce apoptosis and inhibit tumor growth of pancreatic cancer xenografts (Bondar et al., 2002). Our recent data have demonstrated that specific pharmacological inhibition of p110γ using selective inhibitors reduces proliferation of PDAC (Edling et al., 2010) and HCC cell lines (Dituri et al., 2012) *in vitro*. Interestingly we also reported that caffeine and its analog CGS 15943 block proliferation of HCC and PDAC cell lines by inhibiting the PI3K/Akt pathway (Edling et al., 2014). More specifically, a kinase profiling assay revealed that CGS 15943 targets p110γ therefore this study identified this compound as a promising lead compound to develop drugs that can specifically target this PI3K isoform in cancer (Edling et al., 2014).

## Concluding remarks

**Figure 1:**
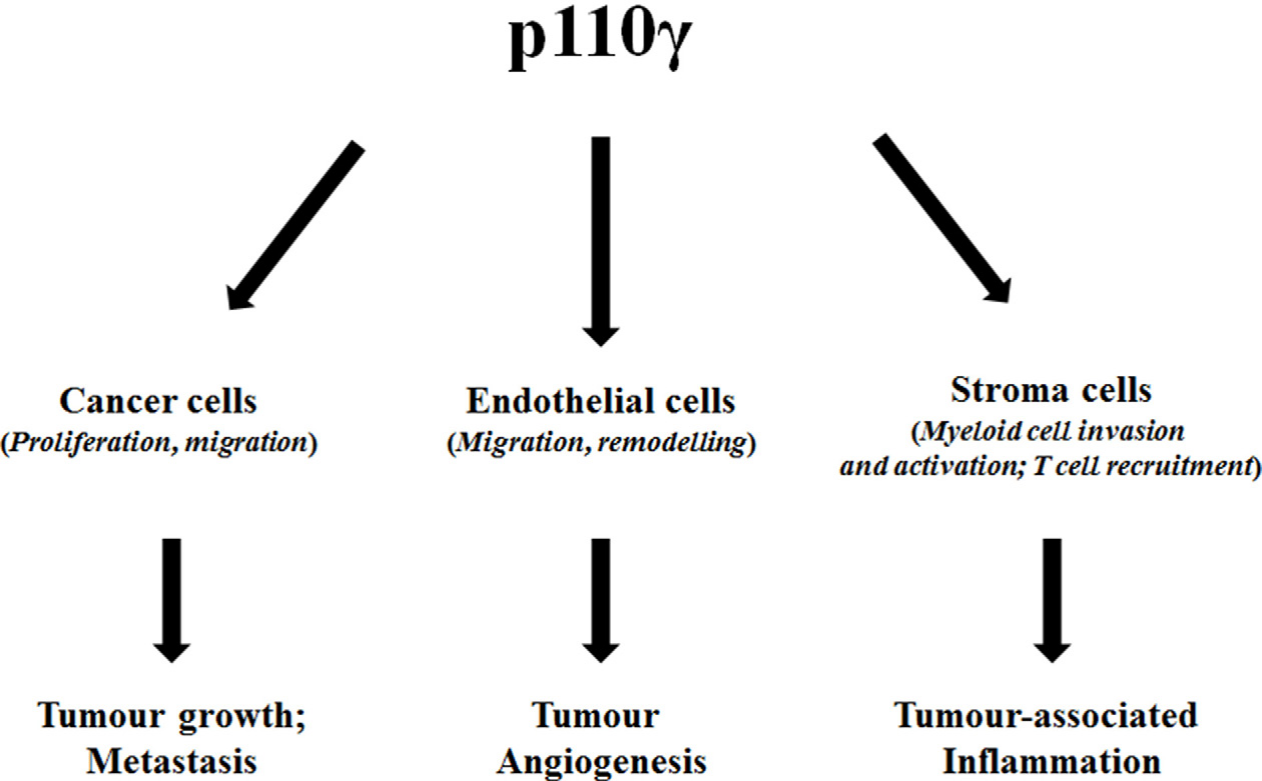
**Diagram summarizing the effect of p110γ on different cell types and ultimately on cancer-associated processes**.

Given the predominant expression of p110γ in hematopoietic cells the interest of oncologists on this specific lipid kinase has been mainly focused on hematological malignancies. Nevertheless, the discovery of many solid cancers where p110γ seems to play a key role has clearly indicated that inhibition of this specific isoform can prove beneficial in a larger spectrum of cancer types. Furthermore, p110γ is a valid target in different tumor-related processes such as metastasis, angiogenesis and cancer associated inflammation (Figure 1). Therefore, this evidence underlines the potential of targeting p110γ in cancer, especially in gastrointestinal cancers. Several p110γ–specific inhibitors are available and they have been already used to alleviate symptoms in inflammatory chronic diseases such as rheumatoid arthritis and systemic lupus. Testing the effect of these inhibitors in solid cancers will represent an important future challenge. In particular, due to the central role of p110γ in regulation of the immune system it would be critical to assess whether such a strategy could result in potential side effects of immunosuppression. On the other hand small inhibitors designed to block the catalytic activity of the enzyme would not be beneficial to counteract its kinase-independent functions. Therefore, novel strategies should be developed to identify specific inhibitors that target the p110γ kinase-independent activity. We propose that certain gastrointestinal cancers represent the tumor types of election where the use of p110γ inhibitors can be particularly beneficial.

### Conflict of interest statement

The authors declare that the research was conducted in the absence of any commercial or financial relationships that could be construed as a potential conflict of interest.

## Abbreviations

EC: endothelial cells
GPCR: G-protein coupled receptor
HCC: hepatocellular carcinoma
LPA: lysophosphatidic acid
mTOR: mechanistic target of rapamycin
PDAC: pancreatic cancer adenocarcinoma
PI3K: phosphoinositide 3-kinase
RTK: receptor tyrosine kinase
S1P: sphingosine-1-phosphate.
